# Diversifying Skin Tone Representation in Medical School Dermatology Curriculum

**DOI:** 10.1007/s40670-024-02159-w

**Published:** 2024-10-02

**Authors:** Devin Barzallo, Afua A. Ofori-Darko, Ashley M. Perez, Katherine DiSano

**Affiliations:** 1 Case Western Reserve University School of Medicine, 501 Euclid Ave, Cleveland, OH 44106 USA; 2 MetroHealth Medical Center, Cleveland, OH 44118 USA

**Keywords:** Education, Dermatology, Curriculum, Skin of color

## Abstract

The lack of diversity in medical education contributes to disparities in dermatologic health outcomes, particularly for patients of color. This study evaluates second-year medical students’ comfort in diagnosing conditions in skin of color (SOC) and explores strategies to improve their confidence. We hypothesize that a curriculum enriched with SOC representation will enhance diagnostic skills. Surveys from Fall 2020 and Fall 2022 showed increased student comfort in diagnosing SOC. Fall 2022 students, exposed to more diverse dermatologic photos, demonstrated greater confidence. These findings underscore the necessity of integrating SOC content into medical curricula to address healthcare disparities.

## Introduction

A lack of diversity in medical education is a significant factor contributing to disparities in dermatologic health outcomes [1, 2]. We aim to identify the comfort level of second-year medical students at an allopathic medical school in diagnosing dermatologic conditions in skin of color (SOC) and explore ways to improve their comfort levels to potentially reduce inequities.

Fitzpatrick skin type is used to classify the skin by its reaction when exposed to sunlight and has been used as a proxy for skin color, with Fitzpatrick skin types IV–VI classified as SOC and I–III classified as non-SOC [3]. While other studies have shown a lack of skin tone diversity in medical school curriculum, the impact of integrating a skin of color curriculum within a medical school curriculum on students comfort in diagnosing SOC has not been well explored. We hypothesize that students in the Fall 2022 (F2022) cohort, after receiving the modified dermatology curriculum with greater SOC representation, will report greater comfort in diagnosing dermatological conditions in SOC compared to the students in the Fall 2020 (F2020) cohort with fewer SOC representation.

## Methods

A survey was administered to second-year students after they completed their respective dermatology curricula in the first semester of their second year for the F2020 and F2022 cohorts. There are mandatory in-person small-group problem-based learning classes where lecture material (slides) are directly applied and lectures that can be viewed virtually or in-person. Coding of all dermatology lecture images was independently done by authors (DB, AO, AP) after establishing interrater reliability using Fleiss’ kappa. The authors DB, AO, and AP reviewed all images presented in the six dermatology lectures and classified them as non-SOC or SOC based on standardized reference images [3]. The chi-square test for independence was used to determine the statistical significance of differences in survey responses between the F2020 and F2022 cohorts. After the F2020 audit, dermatology professors were contacted to increase SOC images in their lectures. For problem-based learning, introduction of diverse patients with SOC were introduced.

### Survey

There were 4 multiple-choice questions pertaining to diversity within the dermatologic curricula, with one free response question. The multiple-choice questions were each paired with a corresponding 5-point Likert item assessing the students’ views on diversity within the curricula. Likert-item options for assessing student exposure to diverse skin types included “Strongly Disagree,” “Disagree,” “Neutral,” “Agree,” and “Strongly Agree,” with each option given a numerical value associated with it (“Strongly Disagree,” 1; “Strongly Agree,” 5) (Table 1).

**Table 1:** Exposure to various skin types in dermatology curriculum materials in lecture and IQ (case-based learning) between 2020 and 2022

Question	Year	Strongly Disagree (%)	Disagree (%)	Neutral (%)	Agree (%)	Strongly Agree (%)	*N*	Mean	Chi-square	*p*-value
I was exposed to various skin types in the images shown in the dermatology lecture materials	F2020	5.50%	14.10%	36.80%	34.40%	9.20%	163	3.3	9.53	0.0491
	F2022	1.60%	7.70%	38.50%	37.40%	14.80%	182	3.6		
I was exposed to various skin types in the images shown in the IQ materials	F2020	5.50%	22.10%	35.00%	27.60%	9.80%	163	3.1	10.89	0.0278
	F2022	3.80%	11.00%	35.20%	33.50%	16.50%	182	3.5		

Additionally, we asked students to rate their ability to identify and diagnose dermatologic conditions in diverse skin types. Likert-item options for assessing student ability to identify and diagnose dermatologic conditions in diverse skin types included “Not at all comfortable,” “Uncomfortable,” “Average,” “Comfortable,” and “Very comfortable,” with each option given a numerical value associated with it (“Not at all comfortable,” 1; “Very comfortable,” 5). A free response question was proposed to gain insight into dermatology materials and their ability to prepare students to care for a diverse patient population.

## Results

**Fig. 1:**
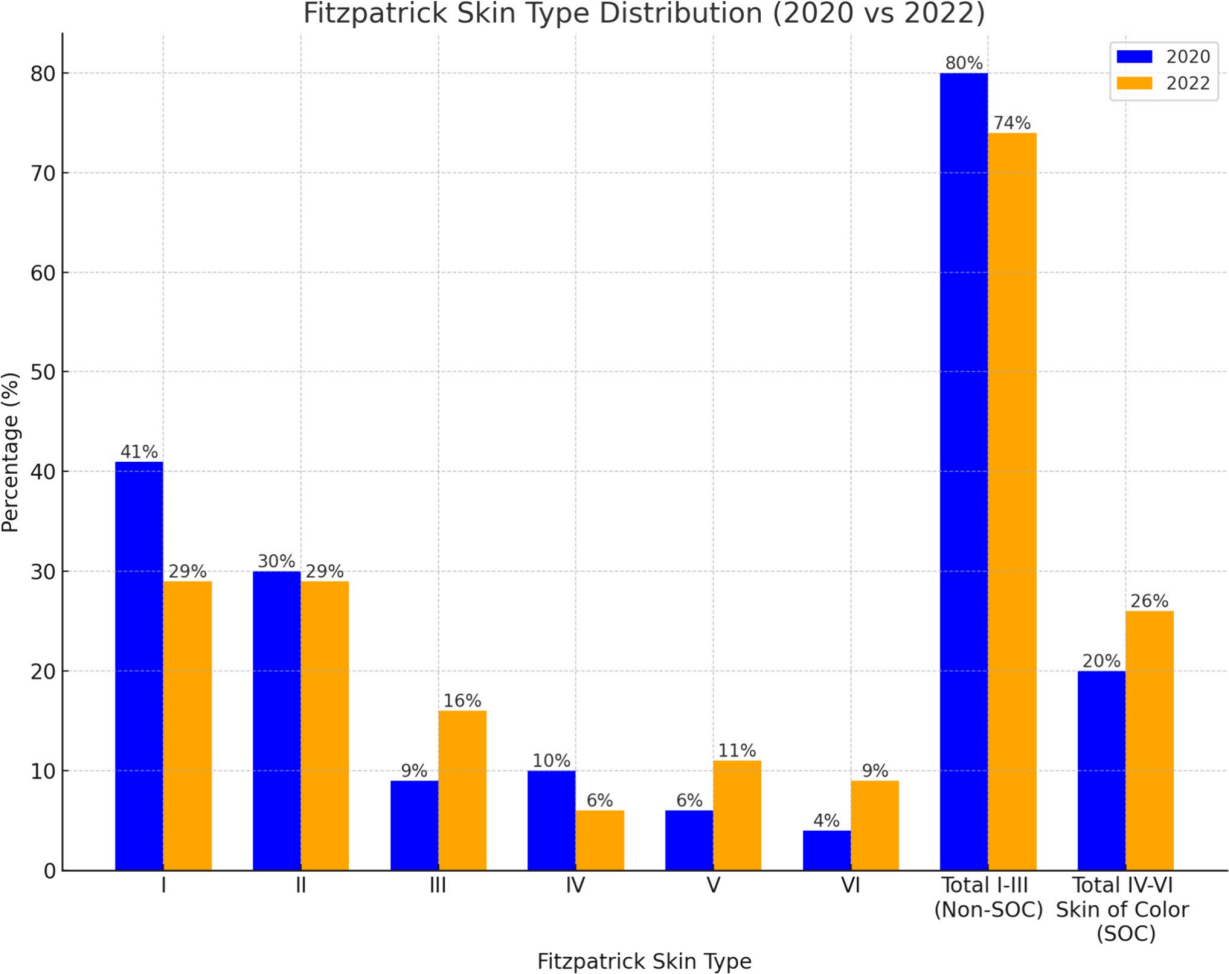
Fitzpatrick skin type (I–VI) representation in dermatologic curriculum at study baseline (F2020) and after curriculum enhancement (F2022). Skin of color (SOC) is identified as Fitzpatrick IV–VI vs non-SOC as Fitzpatrick I–III

Authors (DB, AO, AP) achieved a Fleiss’ kappa of 0.87. The dermatology curriculum audit in F2020 revealed that 63/315 (20%) images displayed conditions in SOC, compared to 100/392 (25.51%) in F2022 (Fig. 1). The response rate for F2020 and F2022 cohorts was 87% (163/187) and 97.85% (182/187). In F2020, 14.1% and 35% of students rated themselves as not at all comfortable and uncomfortable, respectively, in diagnosing conditions in SOC, compared to 4.9% and 21.4% in F2022 (Table 2). In F2020, 43.6% of students agreed or strongly agreed with being exposed to various skin types in dermatology lecture materials, compared to 52.2% in F2022. In F2022, 25.2% felt either comfortable or very comfortable in diagnosing future conditions in SOC compared to 15.4% in F2020 (*p* = 0.00344).

**Table 2:** Comfort levels of students in identifying dermatologic diseases currently and in the future in various skin types between 2020 and 2022

Question	Year	Not at all Comfortable (%)	Uncomfortable (%)	Average (%)	Comfortable (%)	Very Comfortable (%)	*N*	Mean	Chi-square	*p*-value
How comfortable do you feel identifying dermatologic diseases in various skin types?	F2020	14.10%	35%	37.40%	9.80%	3.70%	163	2.5	22.28	0.0002
	F2022	4.90%	21.40%	47.80%	17.60%	8.20%	182	3		
How comfortable do you feel making diagnosis in various skin types in the future?	F2020	12.30%	35%	37.40%	12.30%	3.10%	163	2.6	24	0.0001
	F2022	4.90%	18.10%	51.60%	17.00%	8.20%	182	3.1		

Qualitative feedback from students highlighted a desire for more exposure to educational materials that display dermatological conditions in SOC. In F2020, students acknowledged that there was a lack of representation in dermatology lecture content, and admitted this affected their confidence in diagnosing conditions in skin of color. Specifically, they mentioned they wish they had “comparison photos,” and that “more attempt was made by my classmates to share resources in this area then made by the curriculum.” In F2022, after incorporation of feedback, students reported “These images (SOC) were useful & unique additions to the curriculum. I feel much more comfortable identifying lesions on various skin tones, which I would not have otherwise since third party resources haven’t yet modified their materials to cover darker pigmentation,” and “Overall, I have noticed a great effort in IQ (case-based-learning) materials to represent a diverse patient set.”

## Discussion

These findings highlight the need for improved dermatologic education on skin of color (SOC). The study conducted a needs assessment to identify the comfort level of second-year medical students in diagnosing dermatologic conditions in SOC and explore ways to enhance diversity, addressing the educational gap by incorporating SOC content into the curriculum.

The survey responses indicated that, in F2020, most students did not feel comfortable diagnosing conditions in SOC. However, in F2022, there was a notable increase in students’ self-rated confidence in identifying and diagnosing dermatologic conditions in SOC. This improvement may be attributed to the increased exposure to various skin types in the dermatology lectures, case-based learning using SOC patients, and the introduction of a SOC-specific lecture. The statistically significant increase in confidence suggests that efforts to enhance diversity in the curriculum positively impact students’ knowledge and comfort levels.

The findings of this study align with previous research and highlight the importance of increasing representation of SOC in medical education. Current projected demographic changes in the USA suggest SOC individuals will comprise nearly half the population by 2045. This emphasizes the urgency of addressing the educational gap. Improved dermatologic curriculum, with a focus on all Fitzpatrick skin types, is essential to reducing disparities in dermatologic health outcomes and providing equitable care for patients of color.

Limitations of this study include the survey response rates, which varied between academic years, potentially introducing selection bias. Additionally, the study results represent a single institution, which limits its generalizability. Further research involving a larger sample size among multiple institutions would allow for a more comprehensive understanding of the challenges and potential solutions related to dermatologic education and disparities in health outcomes among patients of color. Further, we aim to investigate if there is a difference in outcomes at in-house exams after the introduction of the SOC curriculum. Ongoing efforts to enhance diversity in medical education are crucial in preparing future physicians to provide equitable care to a diverse patient population and reduce disparities in dermatologic health outcomes [4]. This study demonstrates that even a small increase in SOC representation can impact students experience and comfort in diagnosing SOC.
